# Level of Concordance of Pre-, Intra-, and Postoperative Staging in Cervical Cancers (TREYA Study)

**DOI:** 10.1155/2017/8201462

**Published:** 2017-09-10

**Authors:** M. Toure, A. T. Bambara, K. K. Y. Kouassi, E. N. Seka, J. M. Dia, I. Yao, O. Kimso, I. Adoubi

**Affiliations:** ^1^Oncology Department, Treichville University Hospital, Abidjan, Côte d'Ivoire; ^2^Department of Surgery, Yalgado Hospital, Ouagadougou, Burkina Faso; ^3^Department of Obstetrics and Gynecology, Treichville University Hospital, BP V3, Abidjan, Côte d'Ivoire

## Abstract

Concomitant radiochemotherapy is the therapeutic standard for locally advanced (Ib2 to IVa stage FIGO) cervical cancer. In the absence of a radiotherapy in many of our Sub-Saharan African countries, surgical resection is the only therapeutic method available in hopes of achieving a definite cure. However, criteria for curative surgery are not always met due to preoperative understaging of most of our patients. In addition to socioeconomic factors, the causes for understaging are numerous. These include the lack of personnel or underqualified personnel and the absence of complete workup to assess the resectability of the tumor, but above all the lack of decision-making through multidisciplinary consultation meetings. This study makes a plea in order to provide our hospitals with qualified personnel and adequate technical platform to allow efficient management of our patients with cervical cancer.

## 1. Introduction

Surgery with a curative aim treats not only all the tumor mass but also its subclinical extensions (if clinically there are). This standard method is used in cervical cancer of stage IIA and under [1, 2]. Concomitant radiochemotherapy, on the other hand, has been the therapeutic standard for stages IB2 cancers with tumors mass greater than 4 centimeters to stage IVA since the 2000s [3, 4]. Accuracy in staging is a prerequisite for a successful curative surgery. The International Federation of Obstetrics and Gynecology (FIGO) is the most widely used staging system [5, 6]. However, magnetic resonance imaging (MRI) can help with staging [7, 8].

These different methods can cause discrepancies and errors compared to surgical and pathological staging, errors ranging between 17 and 32% for stage IB and between 50 and 64% for the stages IIB and IIIB [9, 10].

In our countries where the technical platform is limited, very few scientific studies have focused on the degree of concordance between pre-, intra-, and postoperative staging.

The objective of this study was to evaluate the concordances and discrepancies observed between different staging in a cohort of patients operated on for cervical cancer in Sub-Saharan Africa (Côte d'Ivoire and Burkina Faso).

## 2. Patients and Method

This retrospective study was carried out jointly in the Oncology and Gynecology Departments of the University hospital of Treichville and the General Surgery Department of the University Hospital of Yalgado Ouédraogo in Abidjan and Ouagadougou, respectively. We identified 78 patients with histologically confirmed squamous cell carcinoma of the uterine cervix diagnosed between June 1, 2015, and May 31, 2016. The patients were operated on in different health care facilities in these countries. We analyzed the patients' characteristics, the conditions in which the clinical examination and the staging were done, the surgical reports, and the results of the histopathological examination.

### 2.1. Initial Clinical Classification

Patients were examined by one or more medical specialists (gynecologists and/or oncologists) or general practitioners, sometimes under general anesthesia (narcosis). Some examinations (endoscopic and morphological imaging) helped to establish the initial classification according to FIGO (cystoscopy, rectoscopy, pelvic MRI, pelvic CT, and pelvic ultrasound).

### 2.2. Peroperative Staging

The surgical indication was only given after preoperative staging. In every case, the indication was for an enlarged total colpohysterectomy with iliopelvic lymphadenectomy. A surgical report was systematically written after surgery. It assessed the characteristics of the tumor, the degree of parietal and regional infiltration, the number of lymph nodes removed, the ratio of lymph nodes removed/infiltrated, and the quality of the surgical resection.

### 2.3. Postoperative Pathology Staging

All surgical specimens were delivered within 48 hours of surgery to the anatomic pathology laboratories to determine macroscopic characteristics, histological type, number of invaded lymph nodes, resection margins, vascular emboli, and histopathological staging. This staging was done according to the pTNM staging system [11, 12] and the pT category allowed defining the FIGO postoperative classification.

### 2.4. Statistical Analysis

Patient and tumor characteristics were described by the mean, median, and proportion. The concordance between the different stages was evaluated using the Cohen kappa coefficient [13, 14] and represented in Table 1.

Survival was calculated using Kaplan-Meier's method, taking into account the time of participation (in months) and the time between the date of diagnosis and the date of the latest data collected. The follow-up time for patients was 18 months. The latest data were collected from the medical files. For patients who died during hospitalization, the last data recorded were collected from their medical records. Other patients or their relatives were contacted by phone to know their status (“alive” or “deceased”). Survival curves were compared using the Log rank test [15].

The statistical analysis was done using Stata 11 and Epi-Info 3.5.3 software. A threshold of significance of 0.05 was used for analysis.

## 3. Results

Seventy-eight (78) patients with an average age of 43 years met the inclusion criteria (Table 2).

Concordance was poor for preoperative versus postoperative staging with Cohen kappa coefficient at 18.07% (Table 3). On the other hand, it was good (Cohen kappa Cohen 79%) for intraoperative versus postoperative staging (Table 5).

When studying logistic regression analysis, factors related to discordance were found to be multifactorial. In univaried analysis, pain, metrorrhagia, accidental discovery, number of examiners, type of examiner, examination without narcosis, obesity, and failure to perform a CT scan were the overriding factors.

After adjusting other variables, those significantly related to the discrepancy were the type of examiner, the type of morphological examination, and the absence of examination under narcosis (Table 4).

**Figure 1:**
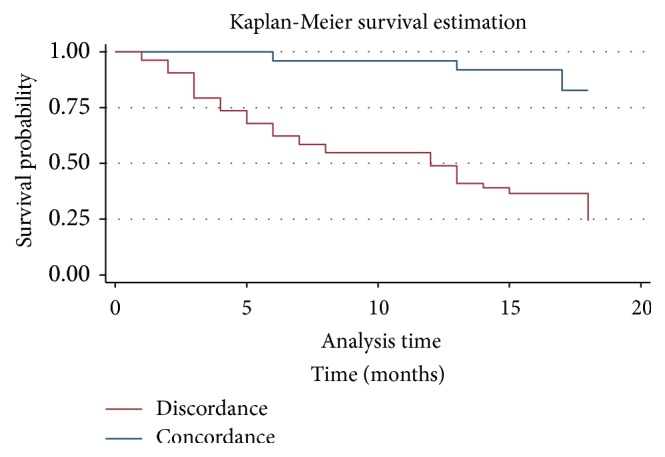
Overall survival: preoperative staging versus intraoperative staging (*p* ≤ 0.001).

**Figure 2:**
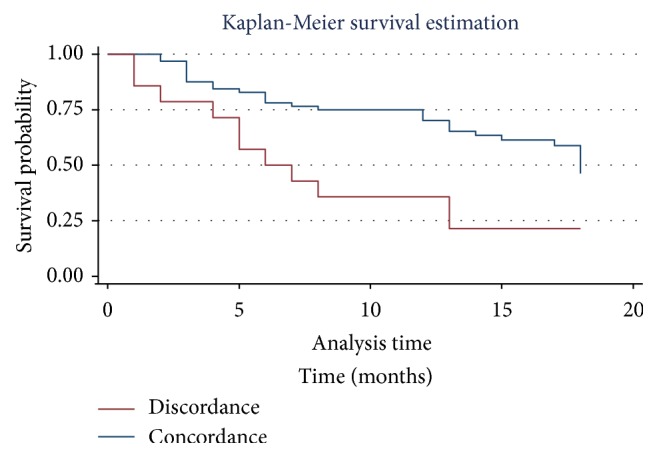
Overall survival: intraoperative staging versus postoperative staging (*p* = 0.0021).

Regardless of the type of comparison (preoperative versus postoperative, intraoperative versus postoperative), survival at 18 months was significantly greater when there was concordance than when there was not (76.5% versus 27.8%, 48.3% versus 19.8%) (Figures 1 and 2).

## 4. Discussion

In Sub-Saharan Africa in general and in Côte d'Ivoire in particular, surgery remains the therapeutic method of reference for cervical cancers [16, 17].

However, this method, in our study for the most part, does not obey the laws of a curative surgery. Indeed, the poor preoperative/intraoperative staging concordance indicates the understaging of our patients. These discrepancies observed in staging could be explained by several factors. They are the inadequacies or the lack of skilled personnel dealing with cervical cancers, the general absence of diagnosis and therapeutic strategy decided during a multidisciplinary team meeting, and, finally, the absence of complete workup to assess the resectability criteria and the operability of the cancer. These various characteristics, which have also been demonstrated in other African series [18, 19], are responsible for frequent postoperative progression with chronic obstructive kidney disease by ureter encasement [18]. Very little data is available in western series due to the paucity of cervical cancers [20]. Moreover, in medicalized countries, the surgical indication is the consequence of an exhaustive pretherapeutic assessment and therefore of care that is similar to the evolutionary stage [21]. However, some western studies have also shown discrepancies ranging from 15 to 20% [9, 10]. The low overall survival rate observed (Figures 1 and 2) is the consequence of these different discrepancies making cervical cancer of bad prognosis [22]. The poor prognosis is related to an inappropriate indication for surgery and thus leading to tumor residue and usually R2 resections.

## 5. Conclusion

For most of our patients, cervical cancer surgery does not meet the criteria of an excision. The various parameters within the framework of the pretherapeutic assessment were insufficiently realized and resulted in a discrepancy between pre-, intra-, and postoperative staging. The stages in which the majority of our patients were consulted were amenable to concomitant radiochemotherapy. The different survival rates were poor, indicating the somber outcome of cervical cancer for most of our patients. This study advocates for efficient management of cervical cancer, which is a model for prevention and healing. Indeed, its risk factors are well known, its natural history is known, and it is curable when detected early.

## Figures and Tables

**Table 1:** 

Kappa coefficient	Level of concordance
>0,81	Very good
0,80–0,61	Good
0,60–0,41	Moderate
0,40–0,21	Mediocre
0,20–0,00	Poor
<0,001	Very poor

**Table 2:** Patients and examination conditions characteristics.

Parameters	Number	Percentage (%)
*Obesity*		
(i) Yes	32	41,03
(ii) No	46	58,97
*Circumstance of discovery*		
(i) Pain	51	65,38
(ii) Metrorrhagia	50	64,10
(iii) Leucorrhea	42	53,85
(iv) Screening	60	76,92
*Performance status*		
(i) 1	50	64,10
(ii) 2	28	36,90
*Nombre of examiners*		
(i) 1	21	26,92
(ii) 2	31	39,74
(iii) 3	26	33,33
*Type of examiner*		
(i) Specialist	45	42,31
(ii) Generalist	33	57,69
*Examination under narcosis*		
(i) Yes	40	51,28
(ii) No	38	48,72
*Patient cooperation*		
(i) Yes	60	76,92
(ii) No	10	23,08
*Pelvic ultrasound*		
(i) Yes	59	75,64
(ii) No	19	24,36
*Pelvic CT scan*		
(i) Yes	38	48,72
(ii) No	40	51,28
*MRI*		
(i) Yes	19	24,36
(ii) No	59	75,64

**Table 3:** Clinical staging versus intraoperative staging.

Preoperative staging	Intraoperative staging							Level of underestimation
	IA	IB	IIA	IIB	IIIA	IIIB	IVA	
IA	**1**	0	0	0	0	0	0	0,0%
IB	0	**10**	4	2	1	0	0	41,2%
IIA	0	0	**10**	5	4	12	3	70,6%
IIB	0	0	1	**2**	2	7	8	85,0%
IIIA	0	0	0	0	**0**	0	1	100%
IIIB	0	0	0	0	0	**1**	2	66,6%
IVA	0	0	0	0	0	1	**1**	**50,0%**

**Table 4:** Factors related to the clinical versus intraoperative staging discrepancies (logistic regression).

	Univariate analysis			Multivariate analysis		
	RC	IC 95%	*p*	RC	IC 95%	*p*
Pain						
No	1			1		
Yes	12,57	[4,1–38,9]	<**10**^−**4**^	4,1	0,6–26,6	NS
Metrorrhagia						
No	1			1		
Yes	11,06	[3,6–33,6]	<**10**^−**4**^	1,1	0,09–13,1	NS
Screening						
No	1			1		
Yes	45,3	[8,9–231,8]		30,5	[4,5–207,2]	**0,0005**
Number of examiners						
1	1			1		
2	0,2	[0,02–1,9]	0,16	0,19	0,02–2,4	NS
3	0,02	[0,002–0,2]	<**10**^−**3**^	0,14	0,007–2,9	NS
Type of examiner						
Specialist	1			1		
Generalist	9,6	[2,5–35,9]		1,4	0,2–11,8	NS
Examination under narcosis						
No	14,3	[3,8–54,1]	**10** ^−**4**^	6,5	[1,3–31,7]	**0,02**
Yes	1			1		
Obesity						
No	1			1		
Yes	25,9	[2,4–33,2]	**10** ^−**3**^	6,9	[1,2–40,9]	**0,03**
CT scan						
No	6,6	[2,1–20,4]	**10** ^−**3**^	0,3	0,05–1,5	NS
Yes	1			1		

**Table 5:** Table of concordance between intraoperative staging and postoperative staging.

Intraoperative staging	Postoperative staging									
	Ia	Ib1	Ib2	IIa	IIb	IIIa	IIIb	IVa	IVb	IV^*∗*^
Ia	**1**	0	0	0	0	0	0	0	0	0
Ib1	0	**0**	0	0	0	0	0	0	0	0
Ib2	0	0	**10**	0	0	0	0	0	0	0
IIa	0	0	0	**14**	0	0	0	1	0	0
IIb	0	0	0	0	**8**	0	1	0	0	0
IIIa	0	0	0	0	0	**7**	0	0	0	0
IIIb	0	0	0	0	0	2	**18**	0	0	1
IVa	0	0	0	0	0	0	0	**6**	0	9
IVb	0	0	0	0	0	0	0	0	**0**	0

*∗* refers to the pathology classifications (pT4) without any “a” or “b” specifications.
